# Binge-eating adolescent treatment (BEAT) – findings from a pilot study on effects and acceptance of a blended treatment program for youth with loss of control eating

**DOI:** 10.1186/s40359-023-01429-3

**Published:** 2023-11-27

**Authors:** Felicitas Forrer, Marius Rubo, Andrea H. Meyer, Simone Munsch

**Affiliations:** 1 Department of Psychology, Clinical Psychology and Psychotherapy, University of Fribourg, Rue P.-A.-de-Faucigny 2, Fribourg, 1700 Switzerland; 2 Department of Psychology, Cognitive Psychology, Perception and Research Methods, University of Bern, Fabrikstrasse 8, 3012 Bern, Switzerland; 3 Department of Psychology, Division of Clinical Psychology and Epidemiology, University of Basel, Missionsstrasse 62A, Basel, 4055 Switzerland

**Keywords:** Loss of Control Eating (LOC), Guided self-help, Blended treatment, Depressiveness, Appearance-based rejection sensitivity, Treatment acceptance, Treatment effects

## Abstract

**Background:**

Loss of Control Eating (LOC) is the most prevalent form of eating disorder pathology in youth, but research on evidence-based treatment in this group remains scarce. We assessed for the first time the effects and acceptance of a blended treatment program for youth between 14 and 24 years with LOC (Binge-eating Adolescent Treatment, *BEAT*).

**Methods:**

Twenty-four youths (mean age 19.1 years) participated in an active treatment of nine-weeks including three face-to-face workshops and six weekly email-guided self-help sessions, followed by four email guided follow-up sessions, one, three, six and 12 months after the active treatment. All patients completed a two-weeks waiting-time period before treatment begin (within-subject waitlist control design).

**Results:**

The number of weekly LOC episodes substantially decreased during both the waiting-time (effect size *d* = 0.45) and the active treatment (*d* = 1.01) period and remained stable during the subsequent 12-months follow-up (*d* = 0.20). The proportion of patients with full-threshold binge-eating disorder (BED) diagnoses decreased and transformed into LOC during the study course, while the abstainer rate of LOC increased. Values for depressive symptoms (*d* = 1.5), eating disorder pathology (*d* = 1.29) and appearance-based rejection sensitivity (*d* = 0.68) all improved on average from pretreatment to posttreatment and remained stable or further improved during follow-up (*d* between 0.11 and 0.85). Body weight in contrast remained constant within the same period. Treatment satisfaction among completers was high, but so was the dropout rate of 45.8% at the end of the 12-months follow-up.

**Conclusions:**

This first blended treatment study BEAT might be well suited to decrease core symptoms of LOC, depressive symptoms and appearance-based rejection sensitivity. More research is needed to establish readily accessible interventions targeted more profoundly at age-salient maintaining factors such as appearance-based rejection sensitivity, while at the same time keeping dropout rates at a low level.

**Trial registration:**

The trial was registered at the German Clinical Trials Register (ID: DRKS00014580; registration date: 21/06/2018).

## Introduction

Loss of control eating (LOC), defined as the sense of loss of control over eating an objectively or subjectively large amount of food [1] is a common subclinical variant of full-threshold binge-eating disorder (BED) in youth (starting at age 15 to 24 years according to the definition of the United Nations, (UN; [2]^1^). Approximately 23% of youth of the general population reported at least one and another roughly 10% at least four LOC episodes during the past month [3], while in weight loss treatment seeking groups nearly 50% reported past or current LOC [4]. There is evidence that even low frequent LOC relates to impaired mental health, increased body weight and an increased risk of developing full-threshold BED compared to no LOC [5–9]. Moreover, subclinical eating disorders such as LOC together with full-threshold BED account for the majority of subject burden and impairment among eating disorders [10]. A recent meta-analysis revealed that LOC remains relatively persistent during the natural course of 15 years in youth of the general population [11]. Therefore, the transition from adolescence to young adulthood may be a period of particular risk for the persistence of LOC [12].

Initial research on the development and maintenance of LOC in youth indicates that interpersonal stressors such as appearance-based rejection experiences interact with deficits in emotion regulation (referred to as interpersonal emotion regulation difficulties), which promotes dysfunctional eating behavior such as LOC [13–16]. Peer acceptance plays a major role in the development of mental health during youth and is substantially influenced by appearance-related characteristics [17]. Youth with LOC are especially concerned about rejection [17] and tend to expect being rejected based on one’s own appearance (appearance-based rejection sensitivity; [18, 19]). To tailor existing treatment programs for BED/ LOC to the needs of youth, interventions targeting interpersonal emotion regulation difficulties such as coping with rejection experiences and appearance-based rejection sensitivity could contribute to a further improvement of treatment effects for young people with LOC.

LOC represents a frequent eating disorder pathology in youth and has detrimental consequences [1], but there is a limited availability and accessibility of adequate treatment resources for both LOC and full-threshold BED in youth [20–23]. In addition, evidence on treatment efficacy for BED is usually based on research in adults, while research on efficacious treatment options in youth with LOC remains scarce.

In adults, psychotherapy represents the treatment of choice for BED [24, 25], with most evidence for cognitive-behavioral (CBT) and interpersonal psychotherapy (IPT). While existing findings indicate that body weight most often remains stable after the treatment of BED or LOC [26] there is evidence that face-to-face psychotherapy (mostly CBT) might outperform CBT-based structured self-help (including unguided and guided respectively online and offline formats) in terms of treatment efficacy of key BED and depressive symptoms and in terms of dropout rates [26–30]. However, structured self-help shows satisfying treatment outcomes and offers the advantage of increasing treatment accessibility and flexibility, especially if provided online [31]. Furthermore, guidance and the online format improve attrition rates in structured self-help for adults [26, 32]. Therefore, structured guided self-help is recommended to reduce core features as a first line treatment of BED and LOC in stepped care approaches [24].

The few existing pilot studies in youth with LOC point to the efficacy of face-to-face psychotherapy as well as of online guided self-help [33–40]. For instance, DeBar et al. [33] found in their study including 26 girls between 12 and 18 years with recurrent LOC, that participating in a face-to-face CBT consisting of eight core and four supplemental sessions decreases LOC episodes and improves shape-, weight-, and eating concerns compared to delayed treatment as usual (TAU-DT). In a clinical trial from Germany, 73 youth aged 12–20 years fulfilling age-adapted criteria of BED (DSM-IV-TR/ DSM-5), including objective and subjective binge-eating episodes, or age-adapted BED of low frequency and/ or limited duration were randomized to an age-adapted CBT consisting of 20 sessions over four months or a four-months waiting-list condition. After treatment, youth who received CBT had substantially fewer monthly binge-eating episodes, achieved higher abstainer rates from binge-eating (51 vs 33%) and remission from BED (57 vs. 33%) as well as lower eating disorder pathology than youth in the waitlist condition. In contrast, CBT showed no specific effects on depression or body mass index (BMI) as the groups did not differ in this regard after treatment. Improvements achieved during the active treatment were sustained during a 24-months follow-up period [39]. Moreover, a study on the efficacy of a 16-week semi-structured online guided self-help CBT with an integrated behavioral weight loss intervention in a sample of 105 overweight male and female high school students at the age of 15 years with LOC found that self-help CBT decreased LOC at posttreatment and nine months later, compared to a waitlist group. Interestingly, youths who received online guided self-help CBT showed lower BMI values as well as a greater reduction of weight- and shape concerns at 9-months follow-up compared to the patients of the waitlist condition [40].

Treatment approaches combining guided face-to-face psychotherapy with the accessibility and flexibility of new technologies such as online structured self-help are named *blended treatments* [41, 42]. Such treatment offers are promising in the treatment of young people as online self-help offers might be more appealing to them and may better meet their typical needs for autonomy and self-determination than pure face-to-face therapy, while close guidance during face-to-face session is still provided. Blended treatments have been shown to be efficacious in depressive youth [43, 44], while there is no such data in BED or LOC research in youth nor adults.

In sum, accessible and appealing evidence-based treatment options for youth suffering from LOC, including interventions on important age-specific maintaining factors such as appearance-based rejection sensitivity, are scarce. Therefore, we developed the first blended treatment program BEAT (Binge-Eating Adolescent Treatment – a training program for adolescents and young adults with LOC), which consists of three face-to-face group or single workshops and six email-guided self-help sessions. The *first goal of the current pilot study*, applying a two-weeks waiting control design, was to assess preliminary effects of BEAT during active treatment and subsequent 12-months (49-weeks) follow-up. We expected the treatment to result in a decrease of the number of patients fulfilling the inclusion criteria of LOC in the present study (at least one LOC episode in the past six months) and BED diagnoses, an increase of the proportion of abstainers from LOC (defined as the absence of LOC during the last month) at posttreatment, and a reduction of the number of weekly LOC episodes during active treatment, compared to the waiting-time period (primary treatment outcomes). We additionally hypothesized that BEAT would result in a decline of the number of depressive symptoms, compared to the waiting-time, and a decrease in average general eating disorder pathology and appearance-based rejection sensitivity between pretreatment and posttreatment (until the end of the active treatment at week 11), but not in body weight (secondary treatment outcomes). We further assumed that these improvements in all these outcomes were maintained during the subsequent 12-month follow-up. Finally, we expected a positive effect of BEAT on average global clinical impression of patients as rated by therapists and patients (secondary treatment outcome).

*As a second aim of this pilot study*, we explored the preliminary acceptance of BEAT in terms of dropout rates and patient’s subjective evaluation of the BEAT program.

## Methods

### Patients

**Fig. 1:**
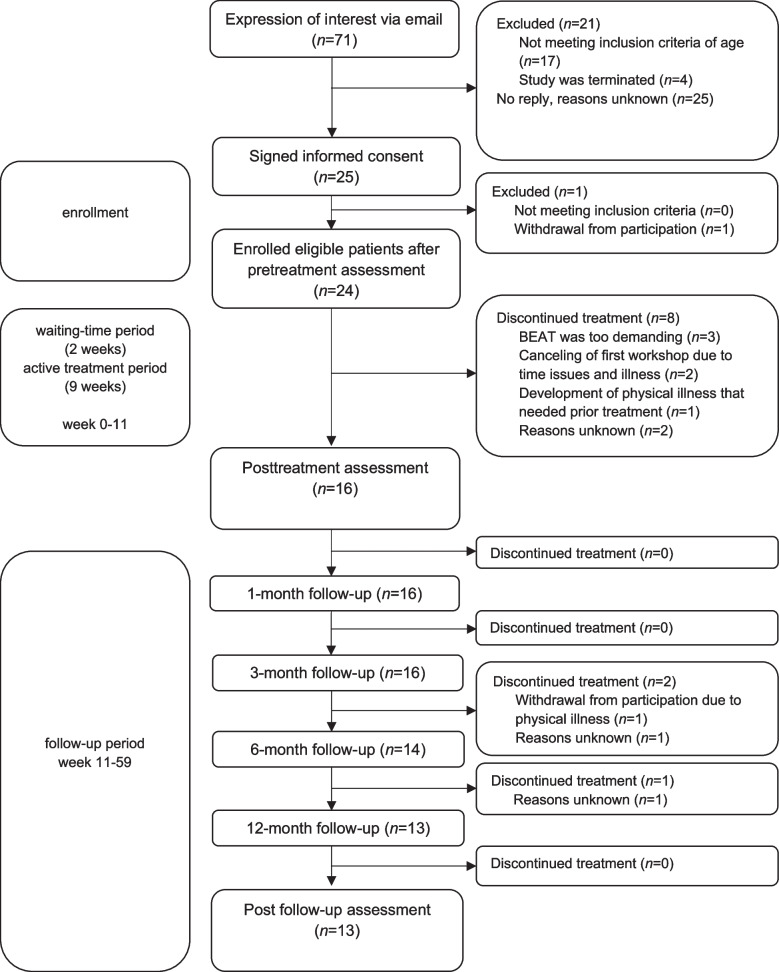
Patient flow diagram

Overall, 24 youths (23 females, one male) were included in the pilot study of whom 16 (all female) completed the posttreatment and 13 the post follow-up assessment of BEAT (see Fig. 1). The recruitment of patients in the present sample took place between May 2018 and February 2020 at the Division of Clinical Psychology and Psychotherapy of the University of Fribourg (Switzerland) and was promoted via public advertisements on webpages and fitness centers, media, as well as cooperating clinicians, healthcare institutions and foundations. The first patient started with the BEAT program in June 2018 while the last patient completed the post follow-up assessment in August 2021. Inclusion criteria were the presence of LOC at least once during the last six months (based on findings that LOC of low frequency is associated with poorer mental health outcomes compared to no LOC; e. g. 7) up to threshold BED according to the DSM-5 [45], age between 14 and 24 years (according to the term youth defined e. g. by the UN [2]) and written informed consent. Exclusion criteria were the presence of another medical or psychological condition requiring prior treatment (e. g. acute substance abuse, psychosis, suicidality), current Bulimia Nervosa or Anorexia Nervosa, pregnancy, the lack of sufficient German language skills, and concurrent participation in a diet or weight loss program or in an eating disorder psychotherapy. As recruiting youth for treatment studies is challenging and since we did not want youth to quit their psychotherapies for participating in this first pilot trial on blended treatment for LOC [33], we did not exclude patients who were receiving concurrent psychotherapeutic treatment for comorbidities, i.e. not eating disorder-specific treatment, from participation in BEAT.

### Study design and procedure

The BEAT pilot study represents a repeated-measures (within-subjects) waitlist control design. After giving informed consent, patients completed pretreatment assessment (week 0) in which online questionnaires were provided and mental disorders assessed in a clinical interview. Thereafter, eligible patients completed a two-week waiting-time (up to week 2; for a detailed overview of study weeks, assessments and study periods, see Table 1) before starting with the first session of the active treatment. The active treatment lasted nine weeks (up to week 11), including nine weekly sessions (one session per week) and the posttreatment assessment, in which the same online questionnaires were provided as for the pretreatment assessment. The follow-up period included 49 weeks (up to week 59) with four follow-up assessments three, 11, 23, and 47 weeks after the posttreatment assessment (i. e. one, three, six, and 12 months after the last session of the active treatment at weeks 14, 22, 34 and 58, respectively), and the post follow-up assessment (week 59).

**Table 1:**
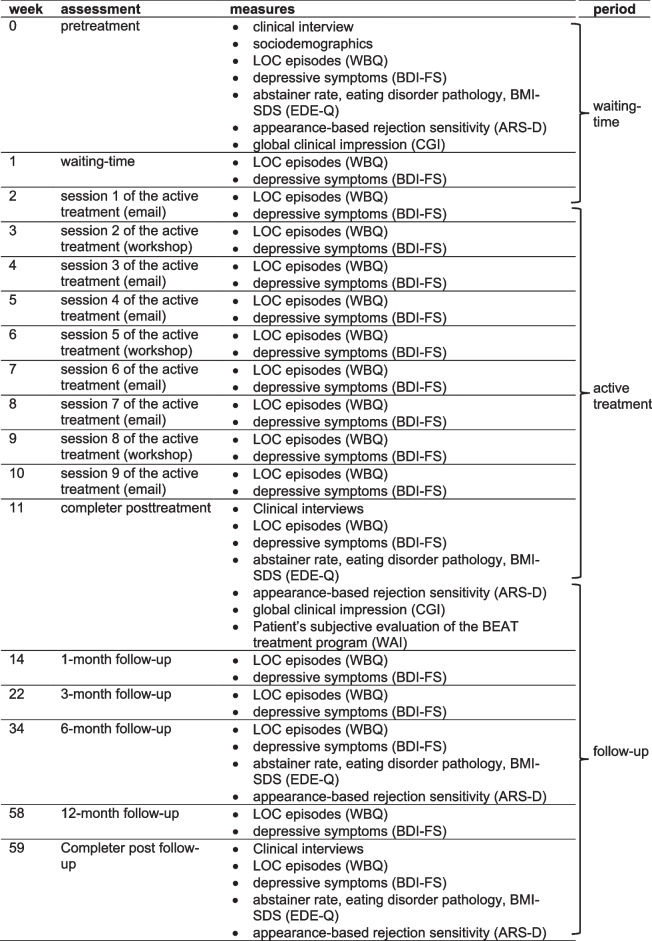
Overview of assessment points and study periods

*WBQ* Weekly Binges Questionnaire, *BDI-FS* Beck Depression Inventory-Fast Screen, *EDE-Q* Eating Disorder Examination Questionnaire, *ARS-D* Appearance-related rejection sensitivity Scale, *CGI* Global Clinical Impression Scale, *WAI* Working Alliance Inventory

Face-to-face workshops during the active treatment were conducted in groups of at most three patients or in a single setting to avoid long waiting times. Of all 24 patients, 12 (50%) participated in the workshops in the individual setting and nine (37.5%) in the group setting. One group comprised three and three groups included two people. Three (12.5%) patients dropped out before the first workshop started. The groups were composed of patients of approximately the same age. Email-guided self-help sessions were processed by patients at home. The BEAT program is based on an evidence-based CBT treatment manual for adults with BED that was developed by our research group [29, 30, 46, 47] and adapted to youth in terms of simplification and adequacy of language and interventions. The treatment further encompasses a training in interpersonal emotion regulation such as coping with rejection and appearance-based rejection sensitivity. During the three workshops that lasted approximately 90–180 min (depending on the workshop and the group- or single setting), all eating disorder specific interventions as well as interventions on interpersonal emotion regulation were discussed and prepared with the corresponding therapist. The email-guided self-help sessions lasted approximately 30–60 min per session and were thought to support patients in implementing interventions that were discussed and prepared during the workshops in daily life. Prior to each email-guided self-help session, patients were provided with a session guide by mail from the therapist, which included information on the goals of the session and age-adapted theoretical background of interventions, links to fill in questionnaires and worksheets with exercises in order to help implementing interventions in daily life. The content of each BEAT session is summarized in Table 2. All treatment sessions were manualized and standardized. However, for some of our youngest patients, the session guides were too demanding and had to be further adapted in terms of length and complexity, without changing the session’s content. To this end, the text in the session guides was reduced and simplified by providing only the basic information needed to understand and perform the corresponding exercises. Furthermore, we had to conduct the workshops online via video call during the first Covid-19 lockdown in Switzerland from end of March to May 2020.

**Table 2:** Main content of the BEAT active treatment sessions

Session	Type of session	Content
1	email 1	introduction into BEAT, LOC and self-observation of eating behavior
2	workshop I	• motivation • BEAT treatment goal • LOC specific CBT - development and maintenance of LOC - regular eating - analyzing LOC episodes with ABC-model - developing coping strategies to overcome LOC (trigger- and reaction control) - working with emergency cards
3	email 2	• individual goal attainment scale and etiological model • typical difficulties in the previous treatment phase • difficulties and improvements in coping with LOC
4	email 3	• difficulties and improvements in coping with LOC • interpersonal emotion regulation (real rejection and appearance-based rejection sensitivity) and its association with LOC • self-observation of situations experiencing real rejection and appearance-based rejection sensitivity
5	workshop II	• difficulties and improvements in coping with LOC • interpersonal emotion regulation: Coping with real rejection experiences and appearance-based rejection sensitivity
6	email 4	• difficulties and improvements in coping with LOC • difficulties and improvements in coping with rejection experiences and appearance-based rejection sensitivity
7	email 5	• difficulties and improvements in coping with LOC • difficulties and improvements in coping with rejection experiences and appearance-based rejection sensitivity
8	workshop III	• difficulties and improvements in coping with LOC • difficulties and improvements in coping with rejection experiences and appearance-based rejection sensitivity • coping with future difficulties
9	email 6	• difficulties and improvements in coping with LOC • difficulties and improvements in coping with rejection experiences and appearance-based rejection sensitivity • individual coping with future difficulties and relapse prevention • further goals
follow-ups	email	• coping with difficulties and relapse prevention

Each patient was assigned to one of four therapists for guidance. All therapists were postgraduate psychologists in CBT training, supervised by SM (principal investigator). After each email-guided self-help session, patients sent their notes, questions and worksheets to their therapist via email. Therapists provided written feedback via email within three days according to standardized topic and text templates (available from the authors) that were derived and adapted for BEAT from previous email- and online based BED treatment programs for adults [30, 48]. All feedback messages were then individualized to the specific needs of the individual patient.

The BEAT pilot study (DRKS00014580; date of registration: 21/06/2018) was approved by the local ethic committee in Switzerland (study ID of the cantonal ethics approval: 2018–00230) and conforms to the Declaration of Helsinki. All patients gave written informed consent prior to their study participation [49].^2^

### Measures

#### *Sociodemographics*

At pretreatment assessment patients were asked to provide age, gender, nationality and occupational status.

#### *Diagnostic interview for mental disorders, short version (Mini-DIPS; *[50]*)*

LOC and further mental disorders were assessed by the Mini-DIPS, a structured interview to assess mental disorders according to the DSM-5 [45], which was conducted by phone before the start of BEAT (pretreatment), one week after the active treatment (posttreatment) and one week after the 12-month follow-up (post follow-up) by the patient’s therapist. Each interview was discussed with FF (responsible PhD student) and SM regarding the diagnoses and the evaluation of inclusion and exclusion criteria. Patients who did not meet full-threshold BED but met the inclusion criterion of at least one LOC episode during the last six months were classified as LOC. The Mini-DIPS has good reliability and validity in outpatient, inpatient and community samples [51, 52].

The following two online self-report questionnaires were assessed weekly during the waiting-time period, before each session of the active treatment, at posttreatment, before each follow-up session, and at post follow-up (total of 17 assessment points).

#### *Weekly Binges Questionnaire (WBQ; *[53]*)*

The WBQ consists of six items that assess the regularity of eating and frequency as well as characteristics of LOC and compensatory behavior during the last seven days. In this study only one single item was included in the statistical analyses to assess the frequency of self-reported weekly LOC episodes by asking youth about the number of episodes in which they experienced loss of control over eating a perceived unusual large amount of food during the last seven days (item 5). It is important to note that self-report assessment of objectively large amounts of food has shown to be less reliable than interview-based assessment [54]. Similarly, the definition of an objective large amount of food and its differentiation from smaller respectively normative food amounts, not fulfilling the criteria of a binge-eating episode according to the DSM-5 [45], is especially difficult in samples with a rather wide age range due to different growth-dependent needs of energy intake and, for younger youth, age-dependent limited access to high quantities of food [7]. Therefore, in youth samples the WBQ assesses LOC (loss of control eating irrespective of the amount of food) rather than binge-eating. At posttreatment and post follow-up, BED diagnoses and the presence of LOC were assessed via phone by the Mini-DIPS and by the WBQ, which was also administered in the interview format. These two instruments allowed to capture core characteristics of full-threshold BED, and of LOC, as well as their duration and frequency. Patients were either classified as presenting a BED, or as LOC, if they experienced at least one LOC episode during the previous six months. At posttreatment and post follow-up, patients who no longer met the diagnostic criteria of at least one binge-eating episode per week during the last three months according to full-threshold BED were classified as LOC and patients who reported no LOC episode during the past six month as “no LOC”. The WBQ shows high convergent validity relative to ecological momentary assessment [55].

#### *Beck Depression Inventory-Fast Screen (BDI-FS; *[56]*)*

The BDI-FS is a short version of the Beck Depression Inventory [57], consisting of seven items and assesses depressive symptoms during the last two weeks. To assess weekly depressive symptoms, we adapted the symptom review period to the past seven days. Sum scores of the BDI-FS range from 0 to 21, with higher scores indicating more depressive symptomatology. The BDI-FS has satisfactory internal consistency (Cronbach’s *α* = 0.84; [58]). Cronbach’s α in the present sample was 0.81 at pretreatment assessment.

The following two online self-report questionnaires were assessed four times (at pre- and posttreatment, 6-month follow-up, and post follow-up).

#### *Eating Disorder Examination-Questionnaire (EDE-Q; *[59]*)*

The EDE-Q assesses eating disorder pathology during the last 28 days. It consists of 28 items, of which 22 items can be assigned to four subscales (*restraint eating*, *eating concern* and *shape*- and *weight concern)* and a global score. For the present study, only the global mean score (i.e. mean of the four subscales) was used, ranging from 0 to 6, with higher scores indicating higher eating disorder pathology. The abstainer rate from LOC was defined as no LOC episodes during the last month, and was derived from item 15 of the EDE-Q, which assesses the number of LOC episodes similarly to the WBQ but during the last 28 days (“Over the past 28 days, on how many days have you eaten an unusually large amount of food and have had a sense of loss of control at the time?”). The EDE-Q was also used to assess self-reported weight and height to calculate age and gender adjusted BMI (kg/m^2^) standard deviation scores (BMI-SDS) according to the LMS method [60]. The EDE-Q subscales and the global score showed good internal consistency with Cronbach’s *α* values ranging from 0.70 to 0.94 [61]. In the present study, Cronbach’s *α* of the EDE-Q global score at pretreatment assessment was 0.93.

#### *Appearance-based rejection sensitivity Scale (ARS-D; *[62]*)*

To assess appearance-based rejection sensitivity, we applied a shortened and adapted version of the German version of the ARS. In the present version of the ARS-D, we chose to present 10 situations, covering topics which are relevant to our age group (the original ARS-D includes 15 situations). For each situation (e. g. “You are trying on clothes in a department store and notice that you have gained some weight in the last week”), patients rated on a 6-point likert scale their appearance-related *rejection concerns* (“How concerned or worried would you be that other people would accept you less/ find you less ok, because of your appearance?”; 1 = very unconcerned, 6 = very concerned) and appearance-related *rejection expectancy* (“How likely would you think it is that other people would accept you less/ find you less ok, because of your appearance?”; 1 = very unlikely, 6 = very likely). For each situation, the score of rejection concern was multiplied with the score of rejection expectation. Then, these scores were used to compute an average total appearance-related rejection sensitivity score across all situations, with higher scores indicating higher appearance-based rejection sensitivity. The ARS-D has satisfying internal consistency with Cronbach’s *α* of 0.90 [62]. The present adapted 10-item version of the ARS-D has not been validated, but Cronbach’s *α* of 0.97 at pretreatment assessment was excellent.

#### *Clinical Global Impression Scale (CGI; *[63]*)*

The CGI assesses the global clinical impression of patients by clinicians before and after treatment, applying three measures: the global severity of illness measure (CGI-S), the global improvement measure (CGI-I) and an efficacy index. The CGI-S, ranging from 1 = not at all ill to 7 = among the most extremely ill patients, was rated at pre- and posttreatment assessment by therapists and additionally by patients. The CGI-I, ranging from 1 = very much improved to 7 = very much worse, was rated at posttreatment assessment by therapists. In the present study, side effects assessed within the efficacy index are reported. Findings regarding the validity of the CGI are inconsistent. While some studies supported the validity of the CGI in clinical trials (e. g. [64]), others criticized the CGI of being inconsistent, biased and too general [65–67].

#### *Patient’s subjective evaluation of the BEAT treatment program (own items)*

Patients reported their satisfaction with BEAT at posttreatment assessment applying eight self-developed items based on the Working Alliance Inventory (WAI; [68], “Overall, how satisfied were you with BEAT?”; “How much did BEAT help you to cope with LOC and the feeling of losing control while eating?”; “Do you think, BEAT is efficacious? “; How much did you like the mix of email-guidance and workshops?”; “How satisfied have you been with the support you received from your therapist?”; “I think, another treatment would have been better for me”; “Would you participate again in BEAT?”; “Would you recommend BEAT to peers?”). Items were rated on a scale from 0 (not at all) to 10 (very much), with higher scores indicating higher satisfaction with BEAT. While each item has been analyzed separately, items were additionally summarized to a mean treatment satisfaction score. Cronbach’s *α* in the present sample of the total score at posttreatment assessment was 0.91.

### Statistical analyses

To report descriptive statistics, we used the mean and standard deviation (*SD*) for continuous variables and absolute numbers and percentages for discrete variables.

Abstainer rates, or, the proportion of patients who were abstinent from LOC after active treatment, were calculated based on the total sample of *N* = 24, and on the sample of completers (including only patients who reached the time point of interest).

The primary outcome WBQ (weekly LOC) and the secondary outcome BDI-FS (depressive symptoms) were assessed regularly (altogether 17 times), thereby allowing us to analyze their weekly temporal course using a discontinuous multilevel model [69], covering three study periods for WBQ and BDI-FS (waiting-time, active treatment and follow-up). The secondary outcomes EDE-Q (eating disorder pathology) and ARS-D (appearance-based rejection sensitivity) covered two study periods (from pretreatment to posttreatment, including waiting-time and active treatment, and from posttreatment to post follow-up). A linear weekly time course was estimated for each phase, resulting in three (WBQ and BDI-FS) or two (EDE-Q and ARS-D) fixed effects. A random intercept and, if this improved model fit, random slope coefficients for the different phases were also included in the model. For the secondary outcome BMI-SDS, no discontinuous multilevel model was set up since previous studies did not point to a decrease in BMI during active treatment (i. e. no specific temporal trend could be specified beforehand). Instead, we set up a multilevel model with time as sole fixed factor (four levels: pretreatment, posttreatment, 6-month follow-up, and post follow-up), plus a random intercept. All analyses were based on the total sample of *N* = 24.

To assess the secondary outcome of the change in the CGI-S scale between pre- and posttreatment, we used the Wilcoxon-test (only completers analysed).

For the WBQ, the Number Needed to Treat (NNT) for a significant treatment outcome during the active treatment relative to the waiting-time was calculated according to Preti [70].

The WBQ was transformed (ln[x + 1]) prior to analyses and predicted means from the multilevel models were back-transformed for reporting. All other outcomes were left untransformed. For continuous outcomes, the effect size *d* was estimated according to Feingold [71] for pre-post designs, i.e. the effect size was based on mean differences between the time points of interest (i.e. pretreatment and posttreatment or post follow-up, respectively) or, with regard to slope differences (waiting-time vs. active treatment or active treatment vs. follow-up), based on the difference of the effect sizes of the time periods of interest. A positive value of the effect size *d* indicates a decrease in the corresponding outcome or a greater overall decrease in mean values in the active treatment compared to the waiting-time.

For analyses related to the second study aim (acceptance of BEAT), we used descriptive statistics, i. e. absolute and relative frequencies or means and standard deviations respectively, to report dropout rates and patients’ subjective evaluation of the BEAT program at posttreatment (completers only).

All analysis were performed using R for statistical computing, version 3.2 [72]. The level of significance was set at 0.05.

## Results

### Sample characteristics

Mean age of the 24 patients (23 females, one male) was 19.1 years (*SD* = 3.5), with 10 patients belonging to the 14–18 years age group (mean age: 15.6, *SD* = 1.8) and 14 patients to the 19–24 years age group (mean age: 21.6, *SD* = 3.2). All patients were Swiss. Seven patients (29.2%) visited secondary school, four (16.7%) were in an apprenticeship, nine (37.5%) were inscribed at a University and four (16.7%) were employed. Sample characteristics at pre- and posttreatment as well as 1-, 3-, 6-, 12-month follow-up and post follow-up are displayed in Table 3.

**Table 3:** Sample characteristics (descriptive values) at pre-and posttreatment, 1-, 3-, 6-, 12-month follow-up and post follow-up

	**Pretreatment total sample (*n* = 24)**	**Pretreatment completer sample **^**a**^** (*n* = 16)**	**Posttreatment (*n* = 16)**	**1-month follow-up (*n* = 16)**	**3-month follow-up (*n* = 16)**	**6-month follow-up (*n* = 14)**	**12-month follow-up (*n* = 13)**	**post follow-up (*n* = 13)**
**Interview-based (Clinical judgment)**								
BED diagnosis, *n* (%) ^b^	21 (87.5)	14 (87.5)	9 (56.3)	–	–	–	–	6 (46.2)
*Mild*	*9 (37.5)*	*6 (37.5)*	*9 (56.3)*					*5 (38.5)*
*Moderate*	*10 (41.7)*	*7 (43.8)*	*0*					*1 (7.7)*
*Severe*	*2 (8.3)*	*1 (6.3)*	*0*					*0*
*Extreme*	*0*	*0*	*0*					*0*
LOC, *n* (%) ^c^	3 (12.5)	2 (12.5)	7 (43.8)	–	–	–	–	6 (46.2)
No LOC, *n* (%) ^d^	0	0	0	–	–	–	–	1 (7.7)
Patients with at least one comorbid mental disorder (%)	20.8	18.8	12.5	–	–	–	–	7.7
**Questionnaire-based (Self-report)**								
WBQ, *M* (*SD*)	3.29 (1.97)	3.25 (2.05)	1.00 (0.97)	1.56 (1.90)	0.94 (1.34)	1.57 (1.99)	1.15 (1.34)	1.62 (1.76)
EDE-Q global score, *M* (*SD)*	3.57 (1.20)	3.86 (0.91)	2.55 (1.01)	–	–	2.48 (1.49)	–	1.89 (1.19)
BMI-SDS, *M* (*SD*)	1.29 (1.28)	1.02 (1.20)	0.89 (1.09)	–	–	0.95 (1.04)	–	0.97 (1.12)
BDI-FS, *M* (*SD*)	6.00 (3.86)	6.38 (2.83)	3.13 (2.16)	2.94 (2.41)	3.19 (2.69)	2.71 (2.73)	2.15 (2.15)	2.15 (2.30)
ARS-D, *M* (*SD*)	13.61 (9.09)	14.07 (8.64)	10.66 (7.99)	–	–	10.36 (8.12)	–	8.55 (6.15)

BED and LOC diagnoses are based on the Mini-DIPS and WBQ interview

*WBQ* Weekly Binges Questionnaire, *EDE-Q global score* Eating Disorder Examination Questionnaire, global score, *BDI-FS* Beck Depression Inventory-Fast Screen, *ARS-D* Appearance-related rejection sensitivity Scale

^a^ only includes patients who have completed posttreatment

^b^ BED diagnosis according to DSM-5

^c^ includes patients with LOC (at least one LOC episode in the past six months) without meeting criteria for BED diagnosis

^d^ includes patients who no longer present with LOC (i. e. no LOC episode in the past six months)

### Preliminary findings on primary and secondary outcomes

#### *Primary outcomes*

##### BED diagnoses and the number of patients with LOC

BED diagnoses and the number of patients with LOC at each time point are reported in Table 3. As expected, BED diagnoses decreased during the study course, whereas the number of patients with LOC increased.

##### Abstainer rate

Calculations for abstainer rates are based on the full sample of *N* = 24 as well as the completer sample at each time point (see Table 4). As expected, abstainer rates increased during the study course.

**Table 4:** Abstainer rates at each time point

	**Pretreatment (*n* = 24)**	**Posttreatment (*n* = 16)**	**6-month follow-up (*n* = 14)**	**Post follow-up (*n* = 13)**
**Abstainer rate, % (*n*)**				
Based on the full sample (total sample of *N* = 24)	4.2 (1)	8.3 (2)	16.7 (4)	16.7 (4)
Based on the sample of completers only (all patients present at current time point)	4.2 (1)	12.5 (2)	28.6 (4)	30.8 (4)

Abstainer rate was defined as no LOC episode during the last month and was derived from item 15 of the EDE-Q

Table 5 shows estimated means for all continuous primary and secondary outcomes derived from the multilevel models.

**Table 5:** Estimated means of primary and secondary outcomes from the multilevel model at pretreatment, posttreatment and follow-ups

	**pretreatment (*n* = 24)**	**posttreatment (*n* = 16)**	**1-month follow-up (*n* = 16)**	**3-month follow-up** **(*n* = 16)**	**6-month follow-up** **(*n* = 14)**	**12-month follow-up** **(*n* = 13)**	**post follow-up (*n* = 13)**
	**Mean [95% CI]**	**Mean [95% CI]**	**Mean [95% CI]**	**Mean [95% CI]**	**Mean [95% CI]**	**Mean [95% CI]**	**Mean [95% CI]**
**Primary outcome**							
WBQ	2.81 [2.06, 3.74]	0.83 [0.54, 1.17]	0.84 [0.56, 1.17]	0.87 [0.60, 1.19]	0.92 [0.63, 1.25]	1.02 [0.62, 1.52]	1.02 [0.61, 1.54]
**Secondary outcomes**							
EDE-Q global score	3.57 [3.07, 4.07]	2.48 [1.92, 3.03]	–	–	2.13 [1.65, 2.61]	–	1.75 [1.15, 2.35]
BMI-SDS	1.29 [0.80, 1.78]	1.14 [0.63, 1.65]	–	–	1.13 [0.62, 1.65]	–	1.18 [0.66, 1.70]
BDI-FS	6.06 [4.72, 7.39]	2.55 [1.53, 3.58]	2.54 [1.59, 3.49]	2.50 [1.71, 3.28]	2.44 [1.72, 3.15]	2.32 [1.13, 3.50]	2.31 [1.10, 3.53]
ARS-D	13.61 [10.24, 16.99]	10.34 [6.64, 14.04]	–	–	9.03 [5.69, 12.36]	–	7.59 [3.68, 11.50]

*WBQ* Weekly Binges Questionnaire, *EDE-Q global score* Eating Disorder Examination Questionnaire, global score, *BMI-SDS* Body mass index standard deviation scores, *BDI-FS* Beck Depression Inventory-Fast Screen, *ARS-D* Appearance-related rejection sensitivity Scale

##### Weekly LOC episodes (WBQ)

**Fig. 2:**
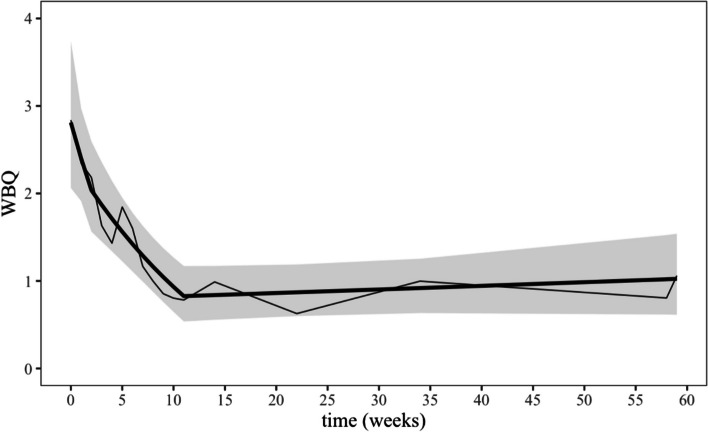
Temporal course of the WBQ from assessment points pretreatment (week 0) to post follow-up (week 59). The line in bold denotes the estimated trendline of the WBQ from the discontinuous multilevel model with turning points set at week 2 and week 11. The values were back-transformed from ln(x + 1). The thin line connects the arithmetic mean of the observed values of the WBQ being present at the different assessment points. These values were first transformed using ln(x + 1), averaged and then back-transformed again to make them comparable to the predicted means form the model-based values. Shaded areas denote ± 1 standard error of the trend lines from the discontinuous multilevel model. Waiting-time: week 0 to week 2, active treatment: week 2 to week 11, follow-up: week 11 to week 59

The WBQ was assessed 17 times during the whole study, which allowed a detailed observation of the temporal course from pretreatment to post follow-up at week 59. Contrary to our hypotheses, the WBQ not only linearly decreased during the active treatment (slope *b* = –0.06, *SE* = 0.01, *t*(272) = –5.02, *p* < 0.001, *d* = 1.01) but also during the waiting-time (*b* = –0.11, *SE* = 0.06, *t*(272) = –1.90, *p* = 0.059, *d* = 0.45; see Fig. 2). The difference between the slopes of these two trendlines was *b* = 0.06 (*SE* = 0.07, *t*(272) = 0.87, *p* = 0.388, *d* = 0.56). Combining these two time periods lead to a linear decrease of *b* = –0.07 (*SE* = 0.01, *t*(202) = –6.92, *p* < 0.001, *d* = 1.53) between pretreatment and posttreatment. The NNT during the active treatment compared to the waiting-time was 3.26. As expected, the WBQ remained stable during the subsequent follow-up period (*b* = 0.002, *SE* = 0.002, *t*(272) = 0.86, *p* = 0.392, *d* = 0.2), the corresponding trendline being less negative than that during the previous active treatment by *b* = 0.06 (*SE* = 0.01, *t*(272) = 4.63, *p* < 0.001, *d* = 1.21). Overall, the difference in the point estimates of WBQ between pretreatment and post follow-up (week 59) was –0.63 (*SE* = 0.14, *t*(272) = 4.61, *p* < 0.001, *d* = 1.26). This refers to a decrease by 1.79 (from 2.81 to 1.02, see Table 5) in the back-transformed values of the WBQ.

#### Secondary outcomes

##### Depressive symptoms (BDI-FS)

**Fig. 3:**
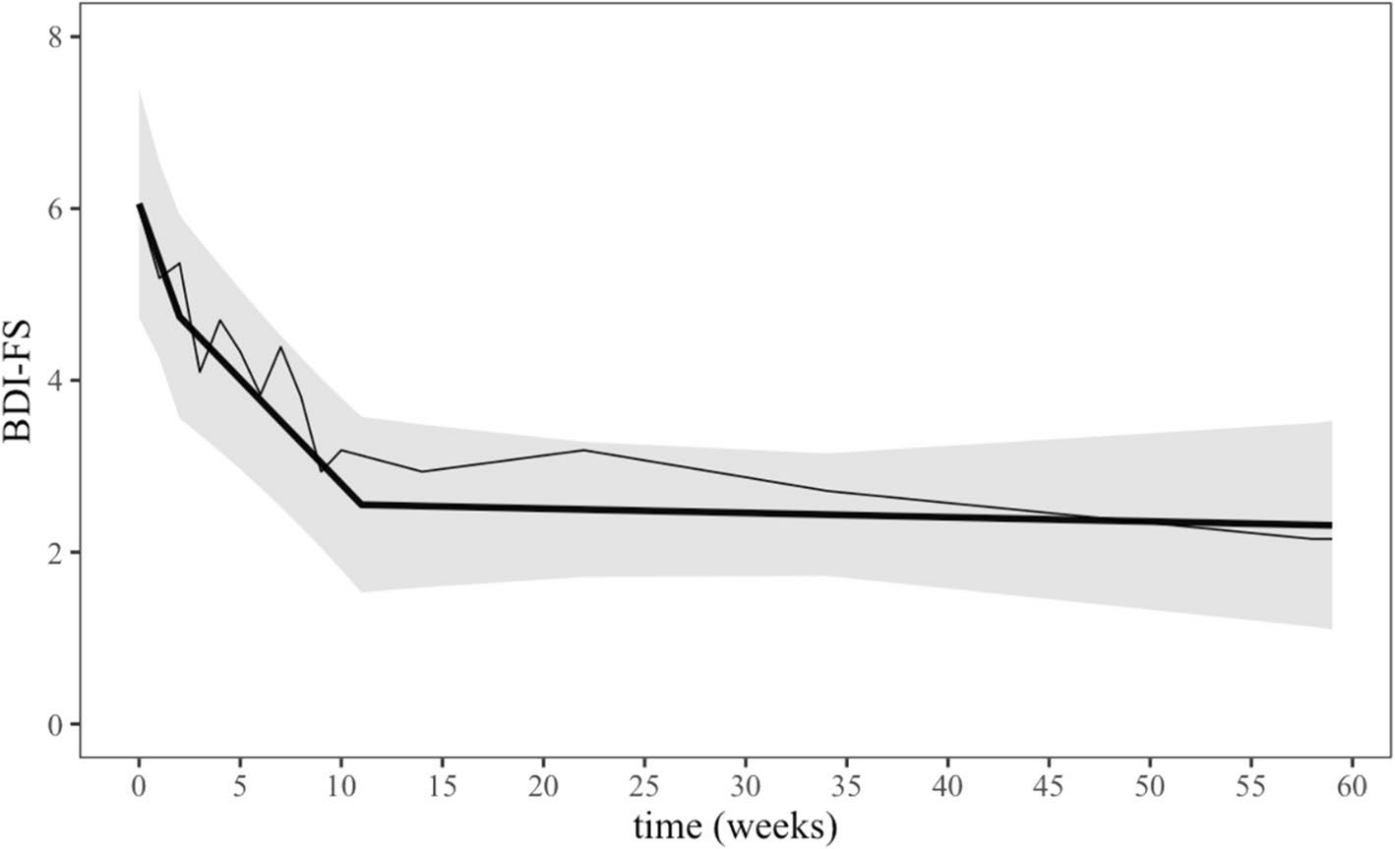
Temporal course of the BDI-FS from assessment points pretreatment (week 0) to post follow-up (week 59). See Fig. 2 for further explanations

As for the WBQ, the BDI-FS was assessed 17 times during the whole study and thus allowed a detailed observation of the temporal course from pretreatment to post follow-up (see Fig. 3).

Contrary to our expectations and similar to the WBQ, the BDI-FS declined during both periods, the waiting-time (*b* = –0.66, *SE* = 0.27, *t*(271) = –2.45, *p* = 0.015, *d* = 0.59) and the active treatment (*b* = –0.25, *SE* = 0.05, *t*(271) = –4.94, *p* < 0.001, *d* = 1.42). The two slopes differed by *b* = 0.42 (*SE* = 0.30, *t*(271) = 1.40, *p* = 0.162, *d* = 0.83). When combining these two time periods, the BDI-FS linearly decreased between pretreatment and posttreatment by *b* = –0.29 (*SE* = 0.07, *t*(201) = –4.43, *p* < 0.001, *d* = 1.50). As hypothesized, the BDI-FS remained stable during the subsequent follow-up period (*b* = –0.02, *SE* = 0.01, *t*(271) = –1.49, *p* = 0.138, *d* = 0.11), the corresponding trendline being less negative than that during the active treatment phase by *b* = 0.24 (*SE* = 0.06, *t*(271) = 4.17, *p* < 0.001, *d* = 1.39). Overall, the decrease in BDI-FS between pretreatment and post follow-up (week 59) was *b* = –3.75 (*SE* = 0.98, *t*(271) = 3.81, *p* < 0.001, *d* = 1.67).

##### General eating disorder pathology (EDE-Q global score) and BMI-SDS

As expected, the EDE-Q global score decreased between pretreatment and posttreatment (week 0 to week 11; including the waiting-time and active treatment) by *b* = –0.10 (*SE* = 0.03, *t*(41) = –3.90, *p* < 0.001, *d* = 1.29) and, contrary to our assumption, also during the follow-up period by *b* = –0.02 (*SE* = 0.01, *t*(41) = –2.20, *p* = 0.033, *d* = 0.85), the slope between pretreatment and posttreatment being significantly more negative than during the follow-up period (*b* = 0.08, *SE* = 0.03, *t*(41) = 2.85, *p* = 0.007, *d* = 0.44). Overall, the decrease in EDE-Q global score between pretreatment and post follow-up was *b* = –1.82 (*SE* = 0.30, *t*(41) = 6.01, *p* < 0.001, *d* = 2.15, see Table 5).

In accordance with our hypothesis, mean BMI-SDS scores did not change between pretreatment, posttreatment, 6-month follow-up and post follow-up (*F*(3, 40) = 0.70, *p* = 0.56, see Table 5).

##### Appearance-based rejection sensitivity (ARS-D)

As hypothesized, the ARS-D decreased between pretreatment and posttreatment by *b* = –0.30 (*SE* = 0.15, *t*(41) = –2.03, *p* = 0.049, *d* = 0.68) and did not substantially change during the follow-up period (*b* = –0.6, *SE* = 0.04, *t*(41) = –1.48, *p* = 0.148, *d* = 0.57). The slope between pretreatment and posttreatment was not significantly more negative than that during the follow-up period (*b* = 0.24, *SE* = 0.17, *t*(41) = 1.41, *p* = 0.165, *d* = 0.11). Overall, the decrease in ARS-D between pretreatment and post follow-up was *b* = –6.02 (*SE* = 1.74, *t*(41) = 3.46, *p* = 0.001, *d* = 1.26, see Table 5).

##### Clinical global impression outcome

Detailed descriptive values of the CGI-S ratings by therapists and patients at pretreatment and posttreatment as well as CGI-I rating at posttreatment by therapists are presented in Table 6. As assumed, CGI-S ratings by therapists (*z* = –3.46, *p* = 0.001) and by patients (*z* = –2.46, *p* = 0.014) were significantly improved at posttreatment compared to pretreatment. No negative side effects of treatment were reported by therapists at posttreatment.

**Table 6:** Outcomes in the clinical global impression scale (CGI)

	**Pretreatment (*n* = 24)**	**Posttreatment (*n* = 16)**
	**Mean (*SD)* /% (*n*)**	**Mean (*SD*) /% (*n*)**
**CGI-S therapist, Mean (*SD*)**	5.17 (0.87)	3.25 (1.00)
not at all ill (1)	0	0
borderline mentally ill (2)	0	25 (4)
mildly ill (3)	4.2 (1)	37.5 (6)
moderately ill (4)	16.7 (4)	25 (4)
markedly ill (5)	37.5 (9)	12.5 (2)
severely ill (6)	41.7 (10)	0
among the most extremely ill patients (7)	0	0
**CGI-S patient, Mean (*SD*)**	4.08 (1.86)	3.19 (1.68)
not burdened at all (1)	20.8 (5)	31.3 (5)
borderline burdened (2)	4.2 (1)	0
mildly burdened (3)	0	12.5 (2)
moderately burdened (4)	16.7 (4)	37.5 (6)
markedly burdened (5)	41.7 (10)	12.5 (2)
severely burdened (6)	12.5 (3)	6.3 (1)
extreme severely burdened (7)	4.2 (1)	0
**CGI-I therapist, Mean (*SD*)**		2.31 (0.60)
very much improved (1)		6.3 (1)
much improved (2)		56.3 (9)
minimally improved (3)		37.5 (6)
no change (4)		0
minimally worse (5)		0
much worse (6)		0
very much worse (7)		0

*CGI-S therapist* CGI severity scale therapist rating, *CGI-S patient* CGI severity scale patient rating, *CGI-I therapist* CGI improvement scale therapist rating

### Preliminary findings on acceptance 

#### Dropout rates

Of the 24 patients enrolled at pretreatment assessment, 11 patients (45.8%) terminated the BEAT program prematurely (dropouts): Of these, one (9.1%) dropped out after the pretreatment assessment in the waiting-time period, seven (63.6%) during the nine treatment sessions of the active treatment, and three (27.3%) during the follow-up period. Considering dropout reasons, for three patient (27.3%) the BEAT program was too demanding, especially the email-guided self-help sessions, two patients (18.2%) cancelled the first workshop due to time issues or illness, two patients (18.2%) had developed a physical illness that needed prior treatment and for four patients (36.4%) dropout reasons were unknown as they didn’t reply to emails and phone calls. Compared to patients who remained in the study, those who terminated BEAT prematurely did not differ with respect to pretreatment values of age, ARS-D, BDI-FS, EDE-Q global score, BMI-SDS, and WBQ (*p* ≥ 0.12 for any of these comparisons).

#### Patient’s subjective evaluation of the BEAT program

Mean treatment satisfaction of the completers (*n* = 16) at posttreatment assessment was 8.86 (*SD* = 1.22) on a scale from 0–10 (0 = not at all satisfied, 10 = very much satisfied). Results of the single item analyses are presented in Table 7.

**Table 7:** Treatment satisfaction with different aspects of BEAT

	**Mean (*SD*)**
Overall, how satisfied were you with BEAT?	8.88 (1.36)
How much did BEAT help you to cope with LOC and the feeling of losing control while eating?	8.44 (1.97)
Do you think BEAT is efficacious?	8.63 (1.45)
How much did you like the mix of email-guidance and workshops?	8.25 (1.95)
How satisfied have you been with the support you received from your therapist?	9.63 (0.72)
I think, another treatment would have been better for me	1.44 (2.16)
Would you participate again in BEAT?	9.25 (1.06)
Would you recommend BEAT to peers?	9.25 (1.29)

## Discussion

*The first goal of the present pilot study* using a within-subject control design was to investigate preliminary treatment effects of the blended treatment BEAT. Participation of youth in BEAT lead to a decrease in the number of BED diagnoses and an increase of patients with LOC. In other words, recurrent binge-eating in our study sample transformed during treatment from BED to LOC (at least one LOC episode during the past six months) at posttreatment and post follow-up (see Table 3). Nevertheless, at post follow-up, only one person experienced no LOC episode during the past six months, which might refer to a limited efficacy of this blended treatment approach and/or illustrates, that subclinical manifestations of BED, such as low-frequency LOC, present for a prolonged period even after treatment. Most of the young people seeking to participate in our treatment study fulfilled the criteria of a BED. This underlines that even though the impact of LOC on physical and mental well-being is already substantial [5–9], youth might seek treatment only when the full picture of BED is present. 

Abstainer rates increased from 4.2% at pretreatment to 12.5% (based on completers only) or 8.3% (based on full sample) at posttreatment and to 30.8% (based on completers only) or 16. 7% (based on full sample) after 59 weeks at post follow-up. These estimates at posttreatment thereby lie distinctly below aggregated estimates from face-to-face (c. 50%) and online structured self-help treatments (c. 45%, mostly based on CBT) in adults [26, 73]. The percentage of youth who does no longer experience LOC since 28 days in our study is further lower than the abstainer rates of LOC/ binge-eating of approximately 92% [33] and 50% [39] as reported in two face-to-face treatment studies in youth with LOC, three respectively four months after treatment start. Compared with previous studies of our group on BED in adults, the abstainer rate after BEAT was lower than in a face-to-face group setting (39%, [29]), but comparable to that from our recent email-guided self-help program (15%, [48]). Such discrepancies in abstainer rates among studies have also been found in the meta-analysis of Hilbert et al. [26] on face-to-face therapy and structured self-help treatments for adults and are based on differences in treatment length, definition of abstainer rates, and statistical concepts (e. g. intent-to-treat versus completer analysis) being applied. For instance, DeBar et al. [33] defined abstainer rate from LOC as zero LOC episodes at posttreatment, while we defined it as zero LOC episodes during the last 28 days according to treatment studies in adults [73]. In the study of Hilbert et al. [39], treatment duration was substantially longer than in BEAT. Moreover, DeBar et al. and Hilbert et al. investigated face-to-face treatments that are so far known to be slightly more efficacious compared to structured self-help [30, 33, 39]. 

The number of weekly LOC episodes decreased during the waiting-time and during the active treatment. Marked differences in the length between the waiting-time and active treatment (2 weeks vs. 9 weeks) might explain why the temporal declines of weekly LOC episodes were comparable between these two study periods. In this context, we would like to point out that it would not have been feasible to make our patients wait for more than two weeks before starting treatment. In addition, as this was the first blended treatment study in minors in Switzerland, ethical concerns regarding waiting-time for minors were prioritized over methodological arguments. Youths are generally hesitant regarding treatment participation [20, 74] and therefore after signaling interest in participation had to be included and transferred to treatment relatively rapidly. We therefore accepted the disadvantage that waiting period of two weeks was likely too short to reliably assess the course of LOC episodes or depressiveness in the absence of treatment. The observed improvements occurring already while waiting are in line with previous studies [48] and might partly be due to fluctuating episodes of abstinence and recurrence during the course of LOC [75] and by the activation of common treatment factors, such as hope for successful treatment [48, 76, 77]. Nevertheless it has to be considered, that in a recent online guided self-help program for adults with BED [30], we observed no reduction of binge-eating episodes during the four-weeks in the waitlist condition compared to the immediate treatment. In this latter study of our group, the knowing they were assigned to a waitlist group while others directly start the treatment, might have canceled out potential hope induction effects during waiting, while in the present study, all patients were waiting before starting two weeks later. 

Even though the comparable temporal decrease of weekly LOC while waiting or being treated for two weeks has to be considered, when interpreting this study’s findings, the calculation of the number needed to treat, NNT, revealed that 3.26 young people had to be treated during the active treatment period in order to outperform the effect of keeping one young person during waiting for two weeks, which refers to a considerable treatment effect [78, 79]. Moreover, and consistent with the decline in BED diagnoses and the increase in abstainer rates, the evaluation of the effects of the overall study course from pretreatment to posttreatment (*d* = 1.53, combining waiting-time and active treatment), and that from pretreatment to post follow-up (*d* = 1.26) revealed a strong effect with respect to the reduction of the number of weekly LOC episodes. In contrast, during the follow-up period the number of LOC episodes per week remained remarkably stable, which confirmed our expectation that the reduction of weekly LOC episodes was maintained in adolescents’ daily life up to 12 months after the end of treatment.

Depressive symptoms strongly decreased during the active treatment but started already to ameliorate during the waiting-time, which again points to a potential positive expectation and that mood and LOC are strongly related and might influence each other during the treatment course [75]. In line with an online guided self-help program for adults with BED [30] and in contrast to the same program applied as book-based guided self-help [48], depressive symptoms strongly decreased during BEAT from pretreatment to posttreatment (*d* = 1.50, including waiting-time and active treatment) and remained stable during the follow-up period. This resulted in an overall treatment effect on depressive symptoms (pretreatment to post follow-up) of *d* = 1.67. While improvements in depressive symptoms in youth with LOC have also been reported in the face-to-face CBT by DeBar et al. [33] and Hilbert et al. [39], youth participating in the only existing online guided self-help CBT, where treatment was less structured than in our and DeBar’s study, did not benefit [40]. However, based on the finding of Hilbert et al. [39] that depressive symptoms improved comparably in youth receiving CBT and in youth awaiting treatment, the specificity of the improvement of depressiveness after BEAT treatment has to be confirmed. 

In line with most findings from previous research on short-term treatment effects (e. g. [26, 29, 30, 33, 39, 48]), general eating disorder pathology (restraint eating, eating concern as well as shape- and weight concern) was strongly reduced between pretreatment and posttreatment assessment. It was further decreased, although to a smaller extent, during follow-up (total effect *d* = 1.81 for the reduction in general eating disorder pathology between pretreatment and post follow-up). 

The treatment study BEAT included interventions targeting interpersonal emotion regulation to cope with rejection and appearance-based rejection sensitivity for the first time. Appearance-based rejection sensitivity moderately improved between pretreatment and posttreatment, while showing minor improvements during the subsequent follow-up. Considering the whole study period from pretreatment until post follow-up, strong improvements in appearance-based rejection sensitivity were achieved, even though in our study, the training to cope with rejection experiences and appearance-based rejection sensitivity was only initiated in the second half of the active treatment. Considering that appearance-based rejection sensitivity represents an enduring and trait-like disposition in the maintenance of LOC [19], the present improvement might be expected to be even higher in more intensive and prolonged interventions on appearance-based rejection sensitivity. 

As expected, and in line with current research in the treatment of binge-eating and LOC [26, 30, 33, 39], BMI-SDS remained stable. The maintenance of BMI-SDS can be interpreted as a successful treatment outcome considering the trajectory of continuous weight gain in untreated youth (e. g. [80]). 

We further assessed the therapist and patient’s impressions of general improvement by applying the CGI. The severity of illness in the CGI-S decreased from markedly ill at pretreatment to mildly ill at posttreatment based on therapists’ ratings, and from moderately burdened to mildly burdened based on youth’s ratings. Further, neither therapist nor patients reported negative treatment side effects. In line with a study that investigated how therapists’ CGI ratings correspond with patient’s ratings [81], therapists reported higher impairment than patients themselves. The discrepancy between patient and therapist ratings might be explained by the lack of specific language use in CGI items and therefore therapists and patients might refer to different correlates of impairment and also evaluate the importance differently. 

Our *second study aim* was to investigate the acceptance of the BEAT blended treatment with respect to dropout rates and patient’s subjective evaluation of the program. The dropout rate of 45.8% at follow-up lies somewhat above the large range that can be expected from online self-help treatments in eating disorders in adults (6–40%; [31]). Previous treatment studies on LOC in youth showed that a substantial part of youth did not attend all treatment sessions [33] nor did they regularly use an online program [40]. In addition, the overall dropout rate of approximately 28.5% in youth participating in different outpatient mental health care treatment studies [82] indicate that attrition in youth with mental disorders is generally challenging.

Apart from the considerable dropout rate obtained in our study, overall treatment satisfaction among patients who completed BEAT was high with a mean value of 8.9 out of 10. Moreover, patients strongly agreed to participate again in BEAT and would highly recommend it to peers.

### Limitations

There are limitations with respect to the findings of the current pilot study. First of all, the sample size was small and our findings need to be reevaluated in larger samples. In order to prevent the risk of early dropout due to lack of motivation, we further kept the waiting period short (two weeks), which contrasts with waiting periods for psychotherapy of up to two months in Switzerland [83]. Together with the lack of an inactive control condition of comparable length, our study design makes it difficult to attribute the treatment effects exclusively to our specific treatment compared to e.g. spontaneous remission. It has also to be mentioned that we included the waiting-time in the overall treatment effect from pretreatment to posttreatment and pretreatment to post follow-up. Accordingly, pretreatment to posttreatment and pretreatment to post follow-up effects cannot be attributed exclusively to the active treatment. In this pilot study, the diagnostic interviews and the assessment in the CGI were not conducted by independent assessors, but by the therapists themselves. Therefore, a certain bias on the diagnoses and the evaluation in the CGI cannot be excluded, especially at posttreatment and post follow-up. We further did not audio-record the diagnostic interviews in this pilot study and therefore no data on interrater reliability can be provided. However, all interviews were applied by therapists with experience in conducting structured interviews and diagnoses and the inclusion criteria of LOC were discussed with the study lead (SM and FF). To our best knowledge there is so far no explicit data on the role of treatment adherence in blended treatments. This might be due to the fact that highly structured self-help treatments such as BEAT consist largely of preformulated treatment contents and online-based support between meetings compared to pure face-to-face therapies. It has also to be taken into account, that the assessment of LOC and body weight were based on self-report, which is less reliable than interview-based assessment or direct measurement of body weight, respectively [54]. Another important limitation relates to the substantial dropout rate in the present pilot study and the fact that a systematic investigation of the reasons and predictors of dropouts was not possible due to the small sample size. In our study sample, seven out of 24 young people (approx. 30%) received additional psychotherapy (but not for eating disorders) while participating in BEAT. Due to the small sample size, the impact of an additional non-eating disorder related treatment on the effect of BEAT cannot be specified. Finally, our findings cannot be generalized on the population of male youth and due to Covid-19, we had to conduct some of the workshops via online video conferences during the lockdown from end of March to May 2020.

## Conclusion

The present pilot study documents initial evidence of the effects of a blended treatment program (BEAT) for youth aged 14 to 24 suffering from LOC, including elements of CBT for eating disorders and interventions to improve interpersonal emotion regulation. Large preliminary treatment effects were found for LOC psychopathology, co-occurring depressive symptoms, appearance-based rejection sensitivity and general eating disorder pathology. Patients who completed treatment with BEAT expressed high treatment satisfaction, but a considerable dropout rate calls for more detailed analyses in attrition to blended and online treatments in this age group. Larger, randomized between-group control designs are needed to more thoroughly test the specific effects of BEAT.

## Data Availability

The datasets generated and/or analyzed during the current study are not publicly available due to sensible data but are available from the last author (simone.munsch@unifr.ch) on reasonable request.

## Abbreviations

ARS-D: Appearance-based rejection sensitivity Scale, German version
BDI-FS: Beck Depression Inventory-Fast Screen
BEAT: Binge-eating adolescent treatment
BED: Binge-eating disorder
BMI: Body mass index
BMI-SDS: BMI standard deviation scores
CBT: Cognitive-behavioral therapy
CGI: Clinical Global Impression Scale
CGI-I: CGI global improvement measure
CGI-S: CGI global severity of illness measure
EDE-Q: Eating Disorder Examination-Questionnaire
IPT: Interpersonal psychotherapy
LOC: Loss of Control Eating
Mini-DIPS: Diagnostic interview for mental disorders, short version
NNT: Number Needed to Treat
TAU-DT: Delayed treatment as usual
UN: United Nations
WBQ: Weekly Binges Questionnaire

## Footnotes

1 Note that in this study, young people at the age of 14 to 24 were recruited in order to cover early to mid-adolescence up to young adulthood. Even though this age range slightly differs from the youth definition of the UN, we use the term youth.

2 The present BEAT pilot study is introduced in the study protocol of the main study “i-BEAT”, which is funded by the Swiss National Science Foundation, SNSF, Grant Number SNSFNr: 100001C_185387; DRKS00023706. Detailed information on the study design of the BEAT pilot study are available from the German Clinical Trials Register, DRKS00014580).
